# Distinctive responses in anterior temporal lobe and ventrolateral prefrontal cortex during categorization of semantic information

**DOI:** 10.1038/s41598-021-92726-7

**Published:** 2021-06-25

**Authors:** Atsushi Matsumoto, Takahiro Soshi, Norio Fujimaki, Aya S. Ihara

**Affiliations:** 1 Center for Information and Neural Networks, National Institute of Information and Communications Technology, and Osaka University, 588-2 Iwaoka, Iwaoka-cho, Nishi-ku, Kobe, Japan; 2 Present Address: Kansai University of Welfare Sciences, Kashiwara, Japan; 3 Present Address: Kyoto University, Kyoto, Japan

**Keywords:** Neuroscience, Psychology

## Abstract

Semantic categorization is a fundamental ability in language as well as in interaction with the environment. However, it is unclear what cognitive and neural basis generates this flexible and context dependent categorization of semantic information. We performed behavioral and fMRI experiments with a semantic priming paradigm to clarify this. Participants conducted semantic decision tasks in which a prime word preceded target words, using names of animals (mammals, birds, or fish). We focused on the categorization of unique marine mammals, having characteristics of both mammals and fish. Behavioral experiments indicated that marine mammals were semantically closer to fish than terrestrial mammals, inconsistent with the category membership. The fMRI results showed that the left anterior temporal lobe was sensitive to the semantic distance between prime and target words rather than category membership, while the left ventrolateral prefrontal cortex was sensitive to the consistency of category membership of word pairs. We interpreted these results as evidence of existence of dual processes for semantic categorization. The combination of bottom-up processing based on semantic characteristics in the left anterior temporal lobe and top-down processing based on task and/or context specific information in the left ventrolateral prefrontal cortex is required for the flexible categorization of semantic information.

## Introduction

Categorization, in which concepts are classified into categories for some purpose, is a central issue in cognitive linguistics and cognitive science. This process is fundamental not only in language but also in interactions with the environment. The semantic concept of a thing (e.g., dog) is characterized by many applicable features (e.g., “has four legs”). The cognitive basis for the categorization of semantic concepts has been investigated and discussed since ancient Greece. The classical Aristotelian model suggested that categories are characterized by a set of features shared by all members. According to this theory, categories should be clearly defined, mutually exclusive, and collectively exhaustive. However, Wittgenstein (1953)^1^ demonstrated that Aristotle’s view cannot explain even simple categories and suggested that members in a category may be connected by a series of overlapping similarities where no one feature is common to all members. Rosch (1973)^2^ and other cognitive scientists suggested that categorization is the process of grouping things based on prototypes, defined as the most central member of a category. These classical views have mainly focused on the organization of semantic concepts in semantic memory systems rather than grouping the semantic concepts into categories, because they considered categories to be based solely on the similarities of semantic concepts. However, categorization is conducted not only based on semantic similarities but also in a highly flexible. For example, a dolphin and a horse are recognized to be in the same category (mammal) in certain contexts, although a dolphin is more similar to fish (e.g. shark) in appearance and other semantic characteristics (features). Therefore, the structure of semantic concepts in semantic memory systems (i.e., semantic similarities between items) alone cannot sufficiently explain these context-dependent categorizations.

Research on perceptual categorical learning has provided important insights on the rational explanation of flexible contextual categorization^3–7^. These studies showed evidence of a multiple model of perceptual categorical learning. While the perceptual learning stage in the visual cortex selectively responds to physical stimulus shape alone, independent of task and context, the stage in the ventrolateral and dorsolateral prefrontal cortex (VLPFC, DLPFC) exhibits a category selective response. In these studies, it was suggested that the visual cortex codes the visual features of stimuli, while VLPFC and/or DLPFC activity reflects learned associations and rules. These findings indicate that the encoding of visual features and contextual categorization of items occur in different neural systems (i.e., different cognitive systems). Additionally, neuroimaging and neuropsychological research has shown the neural dissociation of conceptual information storage and control of these representational systems. Recently, the Controlled Semantic Cognition (CSC) theoretical model^8^ was proposed to explain the relationship between the structure of semantic concepts and flexible contextual categorization. In this model, two interactive neural systems possess the ability to use and manipulate semantic information. The first system encodes the features of concepts, which are widely distributed in the cortex. The second system is responsible for control, manipulating the activation of conceptual systems to generate appropriate behaviors for the specific context. The CSC model assumes that semantic concepts consist of feature information encoded in the modality-specific brain regions, and these modality-specific information sources are reciprocally connected to a single “hub.” For example, the visual representation of a visual object is made by integrating multiple visual features and increases in complexity as the level of processing proceeds^9^. Further, the semantic representation of the object is formed by integrating visual information with information from multiple modalities. The features of these multiple modalities are integrated in the hub to form the representation of a particular object. The hub is assumed to be located within the anterior temporal lobe (ATL)^10–13^. The semantic concepts activated by objects and words must be shaped to align with the context; the VLPFC is thought to be involved in the executive control of semantic concepts^14^. According to the neuroscientific evidence and theories, concepts that share similar semantic features are closely represented with each other, but boundaries for categories can be flexibly drawn between concepts and can change from context to context. This flexible contextual categorization may be established through the appropriate manipulation (by the VLPFC) of semantic information integrated in the hub (at the ATL).

**Figure 1:**
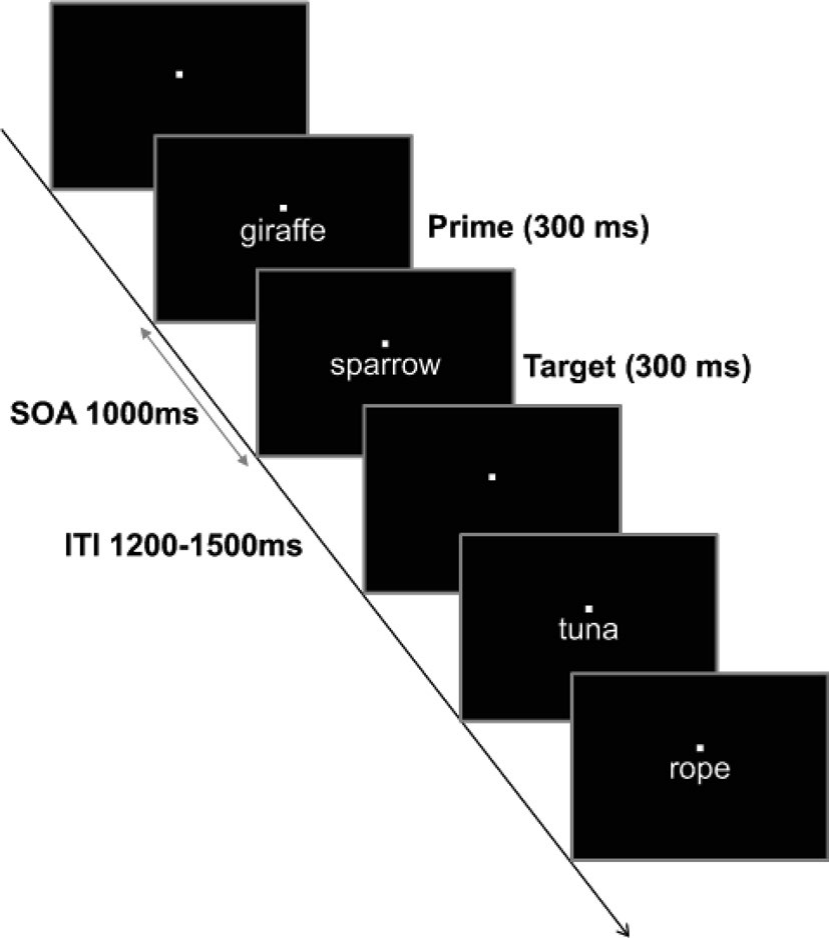
Task scheme of behavioral experiments. Participants were asked to judge whether the target word was living or nonliving as quickly as possible. In the fMRI experiment, the response was required 1000 ms after the presentation of the target word.

In the present study, we conducted behavioral and fMRI experiments using a semantic priming paradigm to examine the multiplicity in contextual categorization of verbal stimuli (Fig. 1). Previous fMRI studies^15–18^ demonstrated that repetition or semantic priming induce neural adaptation (a decrease in neural activity) in various brain regions involved in the processing of target stimuli, particularly in the VLPFC and temporal cortex. The pattern of repetition adaptation enabled us to examine semantic similarity or categorization-related systems when focusing on the relationship between prime and target stimuli.

We used names of animals that belonged to the general category of fish, mammals, or birds for the present study (Table 1). Before the behavioral experiment, the semantic similarities of each pair of animals were investigated based on the subjective judgment of semantic features for all animals. As a result, semantic similarity distance scores between animals were obtained. The group (participants) average distance score was then used as the measure of semantic similarity in the following behavioral and imaging experiments. Subsequently, we examined the behavioral semantic priming effects in the semantic decision task for the animal names. After participants were provided the categories (fish, mammal, bird, and artifact) of the items in the task instructions, they performed semantic decision tasks (living/nonliving judgment task) with target words primed by an animal. Two types of mammals were included: terrestrial and marine. Marine mammals (e.g., dolphins) are unique animals because they share semantic characteristics with mammals, such as viviparity (i.e., embryo development inside the body of the parent), and with fish, such as a marine lifestyle. Investigation of semantic distance based on the similarity of semantic features demonstrated that marine mammals are semantically closer to fish than to other mammals (i.e., terrestrial mammals). Therefore, by focusing on the categorization of marine mammals, we could dissociate the priming effect caused by semantic similarity from the priming effect due to the consistency of the contextual category (i.e., biological grouping into birds, mammals, and fish, appeared in the instructions of the experiment). That is, the semantic priming effect when marine mammals are preceded by terrestrial mammals (within a contextual category), or by animals in the fish category (between contextual categories, but sharing semantic similarities). The same experimental paradigm was used in the fMRI experiment to examine the neural basis of these priming effects.

**Table 1:** Animals used as the stimuli.

No	Fish	TM	MM	Bird
1	carp	Cat	dolphin	chicken
2	goldfish	camel	dugong	crow
3	guppy	deer	eared seal	duck
4	octopus	fox	earless seal	hawk
5	piranha	giraffe	fur seal	ostrich
6	sardine	lion	killer whale	parakeet
7	sea devil	monkey	manatee	pigeon
8	shark	mouse	sea lion	seagull
9	shrimp	sheep	walrus	sparrow
10	tuna	zebra	whale	swallow

TM: terrestrial mammal; MM: marine mammal.

A two-step analysis was performed in the fMRI experiment. First, we addressed the regions that showed semantic similarity dependent activity based on the behavioral ratings of animal features. These regions were thought to be involved in both the processing of semantic representation and contextual categorization. However, it was impossible to dissociate these two processes in the first analysis because the semantic characteristics (i.e., semantic similarity) and contextual categories were not orthogonal. Subsequently, for the regions showing semantic similarity dependent activity, we investigated the priming effects when marine mammals were preceded by fish, terrestrial mammals, or birds. In the second analysis, the regions showing the priming effect concomitant with semantic similarity and the regions associated with contextual categorization could be dissociated because the semantic similarity and contextual categorization were orthogonal in the marine mammal trials.

## Results

The semantic similarity test results showed that marine mammals (MM) were semantically closer to fish than terrestrial mammals, which is inconsistent with category membership.

**Figure 2:**
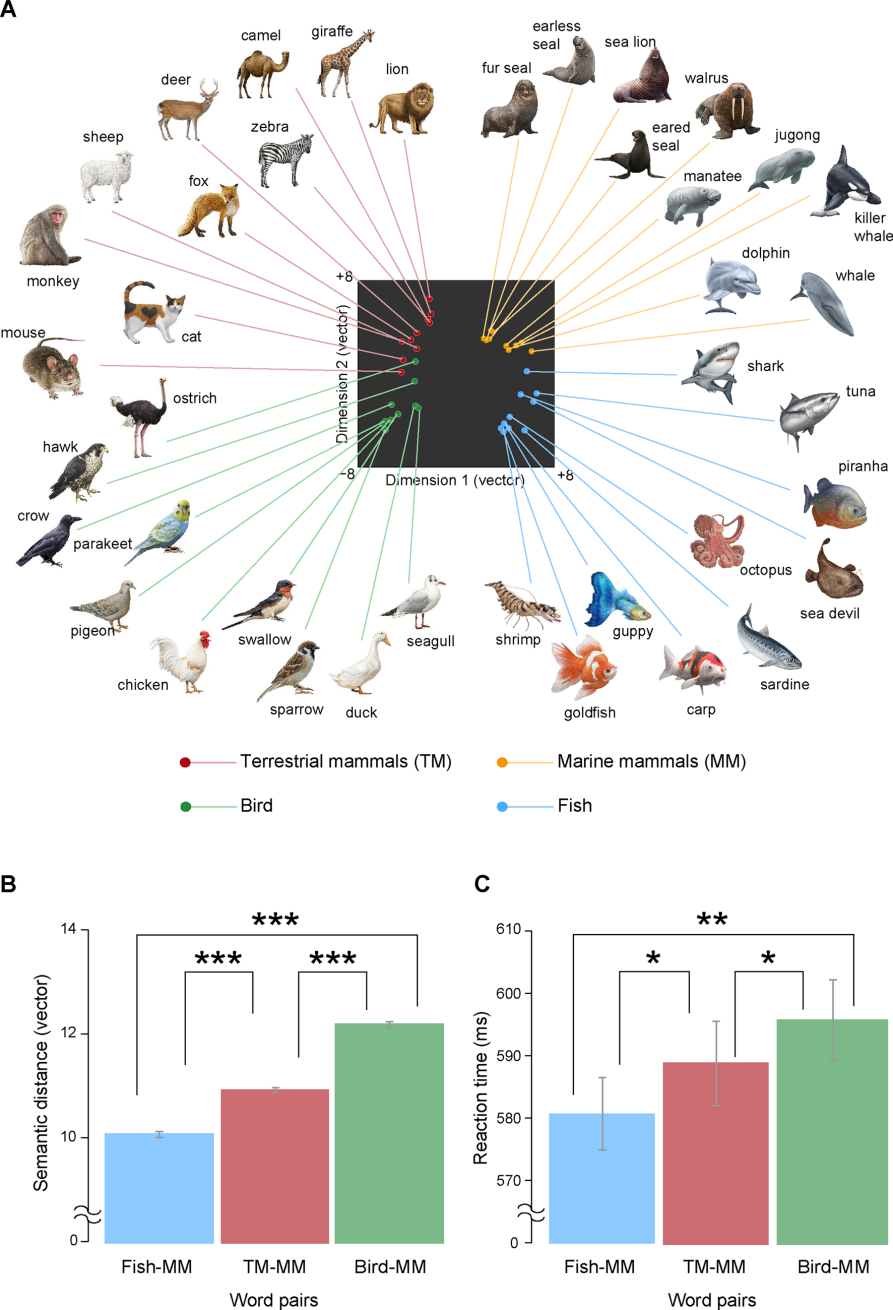
(**A**) Spatial representation of semantic concepts of 40 animals. The semantic similarities between animals are transformed into the spatial distances of reconstructed semantic space by multidimensional scaling. (**B**) Average semantic similarities of word pairs including marine mammals (MM). Semantic similarities of Fish-MM pairs were larger than pairs of terrestrial mammals (TM) and MM, indicating that marine mammals are semantically closer to fish than terrestrial mammals. (**C**) RT of semantic decision for MM target word in behavioral experiments. RTs for Fish-MM conditions (MM target primed by Fish) were faster than those for TM-MM conditions (MM target primed by TM), supporting the notion that marine mammals are semantically closer to fish than terrestrial mammals. Error bars show standard error. *p < .05, **p < .01, ***p < .001. All the illustrations of animals are copyrighted by M/Y/D/S (Yokohama, Japan).

Multidimensional scaling for the data from feature-by-concept applicability judgment test showed the positions of representations of all 40 animals in the semantic space created by the matrix of semantic distance (Fig. 2A). In this space, the first dimension may reflect the animal habitat (land vs. sea) and the second dimension may reflect the animal size or familiarity. The representations of birds, fish and terrestrial mammals were spatially grouped in compliance with their category, whereas marine mammals were closer to fish compared to terrestrial mammals. The average of pairs of each Fish-Marine mammal (MM), terrestrial mammal (TM)-MM, and Bird-MM condition was computed. The mean semantic distance of each condition is illustrated in Fig. 2B. Analyses of variance (ANOVA) revealed a significant main effect of condition (*F*_(2,297)_ = 78.46, *p* < 0.001) and post-hoc tests indicated the semantic distance of Bird-MM pairs was significantly larger than that of Fish-MM (*t*_(298)_ = 11.23, *p* < 0.001) and TM-MM pairs (*t*_(298)_ = 7.55, *p* < 0.001). Further, semantic distance of TM-MM was significantly larger than that of Fish-MM (*t*_(298)_ = 5.30, *p* < 0.001), indicating that participants recognized marine mammals as semantically closer to fish than terrestrial mammals.

In the behavioral experiment, the reaction times (RT) for semantic judgment were recorded and compared between conditions. The results are illustrated in Fig. 2C. ANOVA revealed a significant main effect of condition (*F*_(2,22)_ = 11.44, *p* < 0.001). Post-hoc analysis revealed RTs for the Bird-MM condition were significantly slower than in Fish-MM (*t*_(11)_ = 3.92, *p* = 0.002) and TM-MM conditions (*t*_(11)_ = 3.16, *p* = 0.009). RT for Fish-MM condition was significantly faster than TM-MM condition (*t*_(11)_ = 2.55, *p* = 0.029). These results indicate that the semantic priming effect was larger when marine mammals were primed by fish compared to by terrestrial mammals.

**Figure 3:**
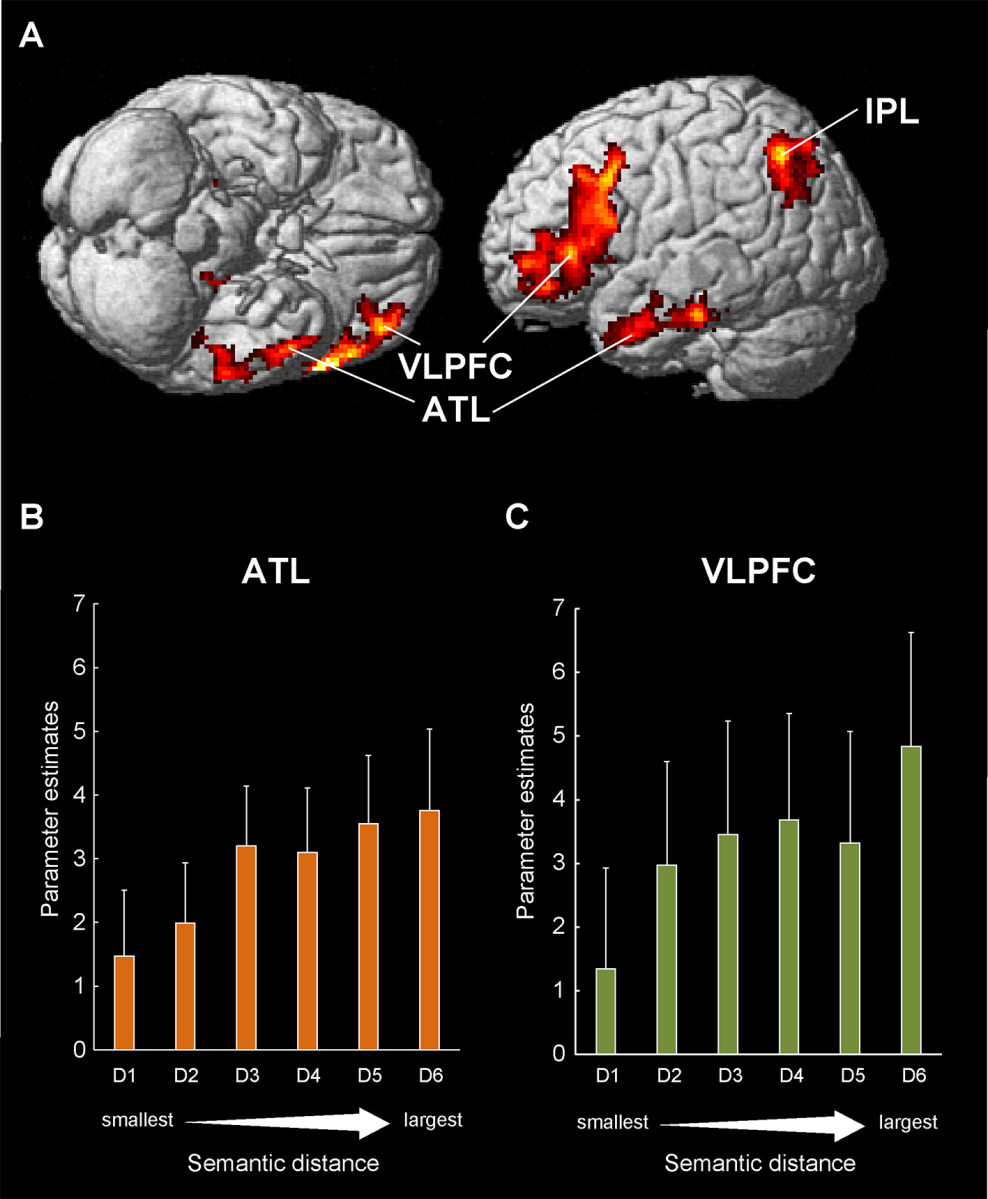
Decreased activation as a function of increasing semantic similarity. (**A**) four regions were detected: the left anterior temporal lobe (ATL), left ventrolateral prefrontal cortex (VLPFC), left inferior parietal lobule (IPL), and posterior cingulate cortex (PCC). All clusters are rendered on the surface of the MNI brain template. All clusters are significant at *p* < .05 after statistical FDR correction for multiple comparisons. (**B,C**) Semantic similarity-dependent activity of the left VLPFC and left ATL. All word pairs are divided into 6 levels (D1-D6) according to the magnitude of semantic similarity and the activations in response to each level are displayed. The activities of these regions decreased as a function of increasing semantic similarity.

For the fMRI data, we first focused on the regions that showed modulation of activity associated with an increasing degree of semantic distance obtained from the semantic similarity test for the prime and target words for all word pairs. Increased semantic distance between prime and target words was associated with increased activity in the left ventrolateral prefrontal cortex (VLPFC), left anterior temporal lobe (ATL), left inferior parietal lobule (IPL), and posterior cingulate cortex (PCC) (Table 2). These regions are displayed in Fig. 3. For the purpose of displaying the pattern of activation, the group mean semantic distances of all word pairs obtained from the semantic similarity test were divided into six bins from small to large distance. The regional activations are displayed for each of the six levels. We found no regions showing increases of activation as a function of decreases in semantic distance.

**Table 2:** Brain areas showing decrease of activation when semantic distance decreased.

Anatomic area	Brodmann area	MNI coordinates			Voxels	Z score
		*x*	*y*	*z*		
L temporal pole	21/38	−50	4	−32	416	4.46
L ventrolateral prefrontal cortex	45	−54	24	8	1833	4.33
Posterior cingulate cortex	31	6	−58	−2	1937	3.99
L inferior parietal lobule	39	−38	−60	42	649	3.69

Activations shown for whole brain analysis. All clusters are significant at *p* < .05 after statistical correction for multiple comparisons　(FDR correction).

**Figure 4:**
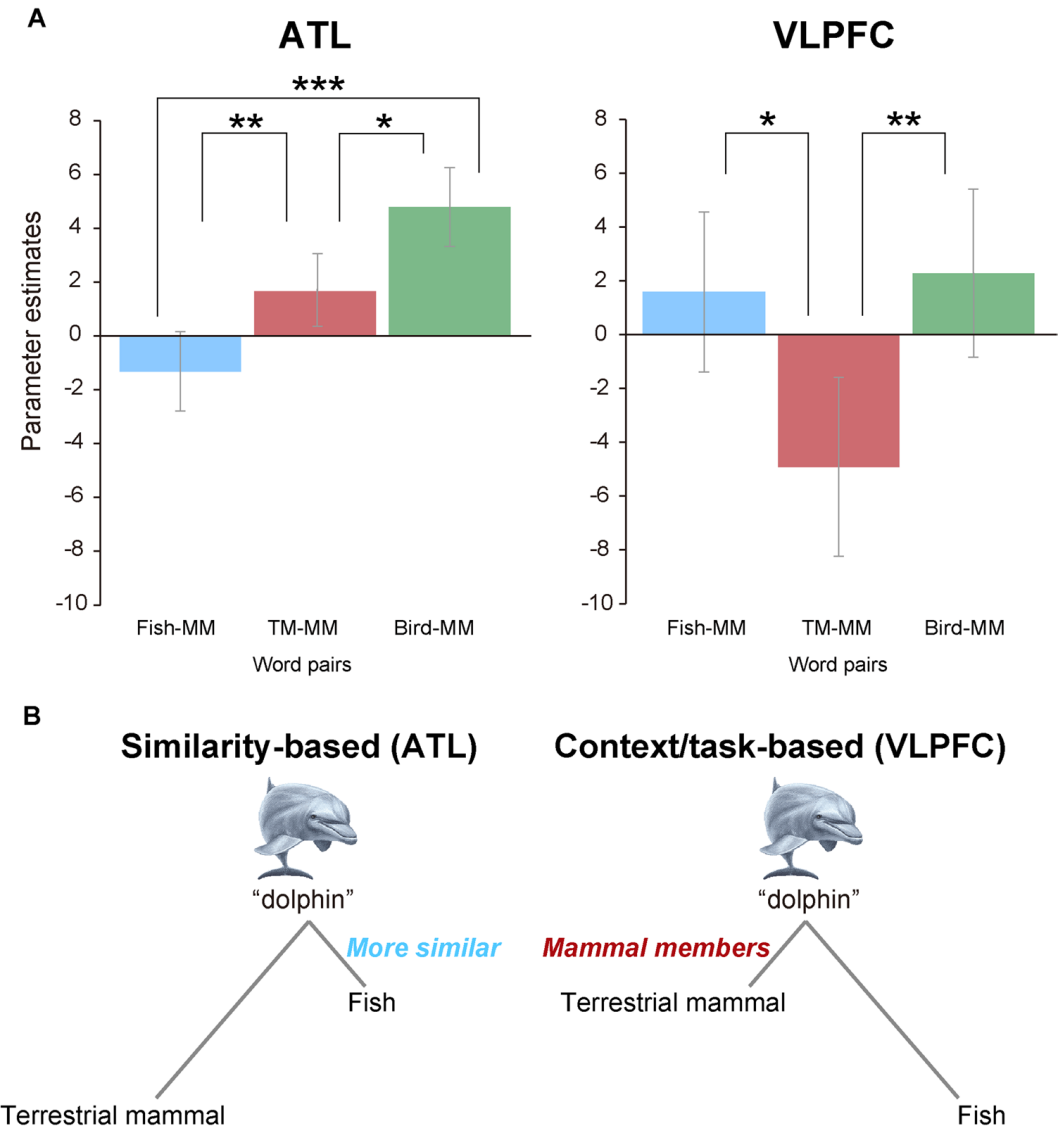
Activations of three MM target conditions in the left ATL and left VLPFC (spherical ROIs centered at the peak voxels of each cluster). (**A**) In the left ATL, the priming effect (i.e., reduction of activation) is observed both in TM-MM and Fish-MM conditions. The priming effect for Fish-MM is larger than that for TM-MM (i.e. priming effects are proportional to semantic distance from MM). In the left VLPFC, the priming effect is shown in the TM-MM condition. (**B**) Conceptual figures represent differential roles between the left ATL and left VLPFC during semantic processing. Dolphins are represented close to fish in the left ATL, whereas they are categorized as mammal in the left VLPFC in this context. Error bars show standard error. *p < .05, **p < .01, ***p < .001. The illustrations of dolphin are copyrighted by M/Y/D/S (Yokohama, Japan).

In general, words belonging to the same biological category had a relatively small semantic distance compared to pairs from different categories. In other words, biological category membership coincides with semantic distance. It is thus unclear whether regions showed increased activity as a function of semantic distance caused by the semantic similarity of word pairs or by the repetition of the words of same category. In this study, we used the unique condition (i.e. MM condition): the items have smaller semantic distances with those in the different category (i.e. Fish condition) compared to those in the same category (i.e. TM condition). Thus, it was possible to examine the detailed patterns of neural adaptation caused by semantic similarity and contextual category assignment for the regions identified in the first analysis. We investigated the priming effect of Fish-MM, TM-MM, and Bird-MM conditions on regions showing significant effect of semantic distance. Two regions, the left ATL and the left VLPFC, showed significant difference of activity in these conditions. Activity is displayed in Fig. 4. There was significant main effect of condition (*F*_(2,30)_ = 22.39, *p* < 0.001) at the ATL. The activation in the Bird-MM condition was significantly larger than in the other two conditions (*t*_(15)_ = 2.85, *p* = 0.012 for TM-MM and *t*_(15)_ = 6.41, *p* < 0.001 for Fish-MM). Activation in the Fish-MM condition was significantly smaller than activation in the TM-MM condition (*t*_(15)_ = 4.75, *p* = 0.001). The left VLPFC also showed a significant main effect of condition (*F*_(2,30)_ = 7.41, *p* = 0.036), but the pattern of activation was different from the ATL. The activation in the TM-MM condition was significantly smaller than other two conditions (*t*_(15)_ = 4.57, *p* = 0.001 for Bird-MM and *t*_(15)_ = 2.59, *p* = 0.041 for Fish-MM). This indicated that this region showed repetition adaptation effect only when the prime and target words belonged to the same category.

## Discussion

In this study, we explored the hypothesis that items sharing semantic features are closely represented with each other, but category boundaries can be flexibly drawn between items and can change from context to context. Our data indicate that semantic similarities represented in the left ATL and the left VLPFC were sensitive to the category membership defined by pre-exposure to three categories.

Our investigation for semantic similarity of word pairs revealed that marine mammals are semantically more similar to fish relative to terrestrial mammals. This indicates that marine mammals are unique items in terms of their inconsistency between semantic characteristics and category. This view was also supported by the results of behavioral experiment. The RTs of the semantic decision for marine mammal target words were faster when primed by fish than terrestrial mammals. According to the spreading activation model of semantic information^19^, in which semantic representations can be easily activated and responses are promoted when the target is preceded by semantically closer representations, this means that marine mammals are semantically closer to fish than terrestrial mammals. Before the experiments, we provided the information about the general category of presented animals (i.e., Fish, Mammal, and Bird), and so participants might observe these categories and temporally form mental sets based on them. However, our instructions concerning the category of the stimuli (i.e., animals in the four biological categories presented in this experiment) did not produce a category related priming effect on RT (i.e., a shorter RT for the TM-MM condition than the Fish-MM condition). In other words, the consistency of contextual category between prime and target word generated no priming effect on RT. This may be because the semantic priming effect mainly affects unconscious semantic processing^20^ and the temporary category effect is a conscious process formed only in this situation.

Numerous studies emphasized the importance of the role of the ATL in semantic processing^10,11,21–25^. Lesion of the ATL causes semantic dementia that exhibits a selective progressive multimodal impairment of conceptual knowledge^24,26^. Schwartz and colleagues (2011) used voxel-based symptom-lesion mapping to explore the likelihood of error lesion distribution in picture naming tasks, and taxonomic errors (“pear” to “apple”) were associated with voxel integrity in the ATL^24^. They concluded the ATL extracts perceptual feature similarity for object processing.

Recent views of semantic processing of words or objects assumed the distributed-plus-hub model in the coding and integration of semantic features^13,27^. According to this model, various conceptual features of objects (e.g. shape, color, smell, and movement) are coded in distributed brain areas, such as perceptual, motor, and emotional regions, and the feature information is integrated in regions called semantic hubs. The ATL is assumed to be an important candidate for this semantic hub^10,11,13,27^. Pobric and colleagues (2010) reported general category impairments after magnetic stimulation of the ATL, whereas stimulation in other regions like the parietal cortex generated category-specific deficits, suggesting the key role of the ATL as a hub in the semantic system^11^. Computationally, generalizable and context-independent semantic concepts were formed at the trans modal hub^28,29^. In this semantic hub model, ATL activity reflects the interaction between the trans modal hub and the distributed semantic features, which establishes the semantic representation. Our results showed sensitivity to the semantic distance between prime and target words in this region. Prior exposure to semantically overlapping representations reduced the activation of semantic features that were shared between prime and target words, which may have reduced the load for the interaction and computation of concepts. Taken together, the ATL activation pattern reflects the semantic similarity of concepts, namely, the architecture of semantic concepts in semantic space.

As a limitation of the present study, we must note that our imaging results may be underpowered with 16 participants; significant effects could not be detected in some regions. Additionally, some ATL activity might be lost by signal distortion. These issues should be addressed in future studies.

The left VLPFC showed an interesting and suggestive pattern for understanding of semantic processing and categorization. This region is associated with various aspects of higher order semantic processing such as retrieval, maintenance, or evaluation of semantic information^30–32^. The left VLPFC showed significantly decreased activity in response to decreases in semantic distance in the parametric modulation of all word pairs. However, precise analysis of the marine mammal target condition revealed sensitivity to contextual category rather than to semantic similarity. This result indicates the left VLPFC is involved in the processing associated with categorization of information, rather than the manipulation of feature information.

The VLPFC plays a key role also in the categorization of perceptual information^4,5,14,33–36^. Neuropsychological studies revealed that VLPFC damage resulted in behavioral impairments in encoding of abstract cognitive variables, such as rules and categories^37^. Freedman and colleagues (2003) showed that after categorization training of monkeys, some VLPFC neurons responded similarly to exemplars from one category and showed lower responses to exemplars from other categories^5^. In an fMRI study on perceptual category learning of human, Jiang and colleagues (2007) showed category-selective activation in the VLPFC^14^. The category selectivity was dissociable from mere shape similarity, consistent with our results. In the present study, although the contextual category (fish, birds and mammals) was not directly related to the task (living/nonliving judgement), participants might form temporary mental sets for categorization, implicitly categorizing items based on the given contextual category.

According to CSC^8^, context-dependent concept structures are assumed to exist within the semantic control systems. Such structures are formed by the integration layer, which interacts with context-independent semantic structures in the supramodal hub and internal context representations. If the VLPFC mediates the formation and maintenance of internal context representation, the observed priming effect in the left VLPFC might reflect the reduced cognitive load for integrating system formation. Alternatively, given that the VLPFC is involved in the selection of semantic features or information^38,39^, the priming effect in the left VLPFC could be explained by the effort required to apply or match features concerning the context-dependent category. Prior exposure to a word in the same contextual category might reduce the cognitive load for the selection and matching of features to a target word.

It may sound strange that the mammal/nonmammal category effect was observed in the left VLPFC when we did not directly require participants to judge whether the target words were mammals or nonmammals. It is reasonable to assume that multiple contextual or task manipulation-related representations can be simultaneously activated. Because the categories of animals (fish, mammals, and birds) used in the present study represent a common and general categorization rule, the participants could automatically form and use these contextual categories, as well as the task manipulation-related representation (i.e., living/nonliving). These results suggest that several context and task representations can be formed and implicitly applied to the encountered items through activity of the left VLPFC. This issue should be assessed by future examination of the priming effect of the VLPFC (and ATL) for multiple tasks (e.g., mammal/nonmammal vs. living/nonliving task). A change in representations across tasks would support this hypothesis.

As other regions (IPL, PCC) identified in the first analysis did not show significant effects in the next analysis, it is difficult to discuss the functional role of these regions. Semantic priming produces changes not only in semantic processing but also in many other cognitive processes by decreasing the cognitive demand for task execution. Given that these regions are thought to be involved in the default mode network^40^ and attentional processing^41^, the significant effects may reflect general difficulty differences independent of semantic processing.

Our data showed two important substrates for semantic categorization. The left ATL showed sensitivity to semantic similarity between the word pairs (i.e. to the structure of the semantic memory system), and the left VLPFC selectively responded to category membership rather than the semantic characteristics of items. These results indicate the existence of a contextual categorization system independent of the structure of representations based on semantic characteristics. Flexible contextual categorization might be impossible if the system was solely based on the structure of semantic representation, processed in an unsupervised fashion and driven by bottom-up stimulus information. Top-down information concerning task- or situation-specific categories, represented in the left VLPFC, may lead to appropriate categorization by interacting with the semantic representation activated in a bottom-up manner. This is consistent with perceptual categorization learning, which assumed the combination of bottom-up and top-down information in occipital cortex and frontal cortex^14,36^.

In sum, our study showed context-dependent sensitivity in the left VLPFC and similarity-dependent sensitivity in the left ATL, suggesting that the semantic categorization is based on two different systems. Our results are helpful to understand the cognitive and linguistic basis of the complex and flexible categorization processing of semantic information.

## Materials and methods

The investigation and experiments in the present study were approved by the human subject ethics committee of the National Institute of Information and Communications Technology (NICT). The approved protocol was in agreement with the Declaration of Helsinki. All methods were performed in accordance with the relevant guidelines and regulations outlined by the human subject ethics committee of the NICT. Participants gave informed consent before the experiments.

### Investigation of semantic similarities

Forty animals were chosen as the stimuli: 10 exemplars of animals each for terrestrial mammals (TM), marine mammals (MM), birds (Bird), and fish, shrimp, and octopus (Fish) so that lexical properties could be controlled among the categories (Table 1). The Kruskal–Wallis test showed no conditional differences in the number of characters, number of morae, or word familiarities (Table 3). The familiarity values on a seven-grade scale were obtained from the Lexical Properties of Japanese database^42^. The word frequencies were obtained from the Balanced Corpus of Contemporary Written Japanese (https://pj.ninjal.ac.jp/corpus_center/bccwj/en/). Although there were significant differences in the word frequency between the TM and MM conditions (corrected *p* = 0.012, Mann–Whitney test with Holm method for multiple comparisons), we selected animals based on the word familiarity, not frequency, as some studies reported that familiarity more accurately explained RT and accuracy rates for word naming than frequency^43,44^. A total of 195 features (e.g. “makes noise”, “runs fast”, “lives outdoors” etc.) were selected from a previous study by De Deyne and colleagues^45^ because it included sample data of feature-concept applicability judgments by four people and overlapped considerably with another feature set^46^.

**Table 3:** Lexical properties of each category.

Lexical properties	Fish	TM	MM	Bird
Number of characters	3.0 (0.9)	2.9 (0.7)	3.5 (0.9)	3.0 (0.8)
Number of morae	2.9 (0.9)	2.9 (0.7)	3.3 (0.9)	2.9 (0.6)
Familiarity	5.9 (0.4)	6.0 (0.3)	5.7 (0.6)	6.0 (0.3)
Frequency (per million words)	3.4 (3.4)	15.3 (18.3)	1.7 (2.8)	6.5 (5.6)

Mean (SD). TM: terrestrial mammal; MM: marine mammal.

Fifteen healthy native speakers of Japanese participated in the investigation of semantic similarities between animals. Participants performed a feature-by-concept applicability judgment test with 7800 pairs (40 animals × 195 features). They were instructed to judge whether the animals had the features by responding either “Yes,” “No,” or “Neither Yes nor No,” respectively, corresponding to score of “1,” “-1,” and “0.” Semantic similarities between all pairs of animals were calculated based on this feature matrix. The measured similarities (δ_*xy*_) were represented by Euclidean distances between paired animals (*x*, *y*) on the basis of response patterns to features (δ_*xy*_ = √ *Σ* (*x*_*i*_ – *y*_*i*_)^2^; *i* = 1,2,…,*n*), where *i* indicates the number of features. In order to project the level of similarity of all animals to semantic space, we applied multidimensional scaling to the distance matrix made by semantic distances between animals, according to the previous study by Caramazza, et al.^47^. The distance matrix was projected to two dimensions. Detailed procedure of data collection of semantic similarity can be referenced to our previous study^48^.

### Behavioral experiment

Twelve healthy, right-handed volunteers (6 females; aged 20–24 years, mean ± SD = 22.8 ± 0.8) participated in behavioral experiments. These participants were distinct from the volunteers who participated in the semantic similarities investigation. All had normal or corrected-to-normal visual acuity. In addition to 40 animals, 20 artificial objects were selected for the sematic judgment task. The experiment involved ten separate sessions; each consisted of 30 experimental and 30 filler pairs. For experimental trials, the target words were marine mammals, which were primed by fish (Fish-MM), terrestrial mammals (TM-MM), or birds (Bird-MM). For filler trials, 5 pairs each of Bird-TM, Fish-TM, TM-Fish, Bird-Fish, TM-Bird, and Fish-Bird were made. Furthermore, 20 TM-artificial object, 20 Fish-artificial object, and 20 Bird-artificial object pairs were used for nonliving trials. Each prime and target word was visually presented for 300 ms. The stimulus-onset asynchrony between the prime and target word was 1000 ms. The intertrial interval randomly varied between 1200 and 1500 ms. Before the experiment, participants were instructed to perform a semantic decision task. The participants were told that two names of animals in four categories (mammals, birds, fish including shellfish, and artifacts) were presented sequentially in a trial and to judge whether the target word was living or nonliving as quickly as possible. To direct participants’ attention to the categories used in this study, we provided the stimulus categories in the oral instructions for the task (e.g., the animals were mammals or birds or fish). We used Presentation software (Neurobehavioral Systems Inc. Albany, CA, USA, https://www.neurobs.com/) for stimulus presentation and recording of participants’ responses and reaction times. Task scheme of experiments are displayed in Fig. 1.

Differences in reaction time were assessed by repeated measured one-way ANOVA among the Fish-MM, TM-MM, and Bird-MM conditions. For planned comparisons, *t*-tests were performed, and alpha levels were controlled by Holm method for multiple comparison.

### fMRI experiment

Sixteen healthy, right-handed volunteers (8 females; aged 20–28 years, mean ± SD = 22.8 ± 0.8) participated in fMRI experiments to investigate neural activity involved in the sematic categorization. These participants were distinct from the volunteers who participated in the behavioral and semantic similarities experiments. All had normal or corrected-to-normal visual acuity. Using all 40 animals and ten artificial objects, 1,960 pairs (100 Bird-Fish, 100 Bird-TM, 100 Bird-MM, 100 Bird-Artificial Object, 90 Bird-Bird, 100 Fish-Bird, 100 Fish-TM, 100 Fish-MM, 100 Fish-Artificial Object, 90 Fish-Fish, 100 TM-Bird, 100 TM-Fish, 100 TM-MM, 100 TM-Artificial Object, 90 TM–TM, 100 MM-Bird, 100 MM-Fish, 100 MM-TM, 100 MM-Artificial Object, 90 MM-MM) were divided into two sets of experimental stimuli. Either set (i.e., 980 pairs) was used for each participant, and sets were equally distributed between participants. The experiment involved ten separate sessions. The stimulus presentation and the instruction for participants were same as those in the behavioral experiment, except for the inter-trial interval (2000–3000 ms) and the timing of response required for the task. In order to eliminate activities of interest (i.e., semantic priming effect) from activities involved in motor control, a swift response was not required in the fMRI experiment. The participants were required to respond after the cue, which was presented 1,000 ms after the onset of the target word, even when the judgment was completed before the cue. Twenty-two null events, in which blanks (4000 ms duration) were presented instead of words, were included in a session to improve the power of estimation.

Functional images of the whole brain were acquired in an axial orientation using a 3 T Siemens Trio MRI scanner equipped with a single-shot EPI (TR = 3 s, TE = 35 ms, Flip Angle = 90˚, 96 × 96 matrix, 37 slices, voxel size = 2 × 2 × 3 mm) sensitive to BOLD contrast. After discarding the first five images, the next 216 successive images in each run were subjected to analysis. An anatomical T1-weighted image was also acquired (MPRAGE, TR = 2 s, TE = 4.38 ms, Flip Angle = 8°, field of view 256 mm, resolution 1 × 1 × 1 mm) for each subject.

### Data analysis

Data were analyzed using Matlab (MathWorks, Inc., USA, http://jp.mathworks.com/) and SPM8 (Wellcome Department of Imaging Neuroscience, London, UK, http://www.fil.ion.ucl.ac.uk/spm/software/spm8/). After correction of head motion, normalization of volumes to MNI space was conducted by use of a transformation matrix which was obtained in the process of normalizing the individual first image to the template, and then spatial smoothing with a Gaussian kernel of 8 mm (full-width at half-maximum) was performed in three axes.

To investigate regions where the activity was modulated by the degree of semantic distance between the prime and target words, we set the degree of semantic distance as a parametric modulator with linear expansion for each target word. In this parametric modulation analysis, we did not model parametric modulations for the trials which were used in the second analysis (i.e., Bird-MM trials, Fish-MM trials, TM-MM trials) to avoid the problem of “double-dipping”^49^. Furthermore, trials that included the names of artificial objects were also excluded from the parametric modulation analysis. The hemodynamic response triggered by the target word was modeled with a hemodynamic response function (HRF), which followed by applying low- and high-pass frequency filters. The activities of Bird-MM trials, Fish-MM trials, TM-MM trials, artificial trials (trials in which artificial objects were presented as a target), and all other trials (general animal trials) were separately modeled, and semantic similarities were entered as a covariate parameter only for the general animal trials. Six contrast images (Bird-MM trials, Fish-MM trials, TM-MM trials, artificial trials, general animal trials, and parametric modulation of general animal trials) were created for each participant. Images were scaled to a grand mean of all voxels and scans within a session^18,50^. The data of parametric modulation for each participant were applied to the random effects model to create a group statistical parametric map. The voxel-wise threshold was set at *p* < 0.01, and clusters were reported if significant at *p* < 0.05 after FDR correction for multiple comparisons across the entire brain volume. Thus, we detected the regions that showed decreased activation as a function of decreasing semantic distance.

In addition, we investigated the semantic priming effect when MMs were processed as a target word. The estimated responses triggered by the target word in Fish-MM, TM-MM, and Bird-MM pairs were entered in ROI analysis for the peak of the regions showing the dependence of semantic distance in the first analysis. The activities were extracted from the 8 mm spherical ROI centered at the peak of each cluster. Differences in the activation of peaks of four regions were assessed using repeated measures one-way ANOVAs among Fish-MM, TM-MM, and Bird-MM conditions. For planned comparisons, *t*-tests were performed, and alpha levels were controlled in all ANOVAs and *t*-tests using the Holm method for multiple comparisons.

## References

[1] Wittgenstein L (1953). Philosophical Investigations.

[2] Rosch E (1973). Natural categories. Cogn. Psychol..

[3] Freedman DJ, Assad JA (2006). Experience-dependent representation of visual categories in parietal cortex. Nature.

[4] Freedman DJ, Riesenhuber M, Poggio T, Miller EK (2001). Categorical representation of visual stimuli in the primate prefrontal cortex. Science.

[5] Freedman DJ, Riesenhuber M, Poggio T, Miller EK (2003). A comparison of primate prefrontal and inferior temporal cortices during visual categorization. J. Neurosci..

[6] Allen L, Mehta S, McClure JA, Teasell R (2012). Therapeutic interventions for aphasia initiated more than six months post stroke: A review of the evidence. Top. Stroke Rehabil..

[7] Op de Beeck H, Wagemans J, Vogels R (2001). Inferotemporal neurons represent low-dimensional configurations of parameterized shapes. Nat. Neurosci..

[8] Lambon-Ralph MA, Jefferies E, Patterson K, Rogers TT (2017). The neural and computational bases of semantic cognition. Nat. Rev. Neurosci..

[9] Kriegeskorte N (2008). Matching categorical object representations in inferior temporal cortex of man and monkey. Neuron.

[10] Holland R, Lambon Ralph MA (2010). The anterior temporal lobe semantic hub is a part of the language neural network: Selective disruption of irregular past tense verbs by rTMS. Cereb. Cortex.

[11] Pobric G, Jefferies E, Lambon Ralph MA (2010). Category-specific versus category-general semantic impairment induced by transcranial magnetic stimulation. Curr. Biol. CB.

[12] Pobric G, Lambon Ralph MA, Jefferies E (2009). The role of the anterior temporal lobes in the comprehension of concrete and abstract words: rTMS evidence. Cortex.

[13] Pulvermuller F (2013). How neurons make meaning: Brain mechanisms for embodied and abstract-symbolic semantics. Trends Cogn. Sci..

[14] Jiang X (2007). Categorization training results in shape- and category-selective human neural plasticity. Neuron.

[15] Dehaene, S. *et al.* Cerebral mechanisms of word masking and unconscious repetition priming. *Nat. Neurosci.* **4**, 752–758. 10.1038/89551 (2001). 10.1038/89551 11426233

[16] Naccache L, Dehaene S (2001). The priming method: imaging unconscious repetition priming reveals an abstract representation of number in the parietal lobes. Cereb. Cortex.

[17] Henson RN (2003). Neuroimaging studies of priming. Prog. Neurobiol..

[18] Matsumoto A, Iidaka T, Haneda K, Okada T, Sadato N (2005). Linking semantic priming effect in functional MRI and event-related potentials. Neuroimage.

[19] Collins AM, Loftus EF (1975). A spreading-activation theory of semantic processing. Psychol. Rev..

[20] Neely JH (1977). Semantic priming and retrieval from lexical memory: Roles of inhibitionless spreading activation and limited-capacity attention. J. Exp. Psychol. Gen..

[21] Lambon Ralph MA, Sage K, Jones RW, Mayberry EJ (2010). Coherent concepts are computed in the anterior temporal lobes. Proc. Natl. Acad. Sci. USA.

[22] Visser M, Jefferies E, Lambon Ralph MA (2010). Semantic processing in the anterior temporal lobes: a meta-analysis of the functional neuroimaging literature. J. Cogn. Neurosci..

[23] Zannino GD (2010). Visual and semantic processing of living things and artifacts: an FMRI study. J. Cogn. Neurosci..

[24] Schwartz MF (2011). Neuroanatomical dissociation for taxonomic and thematic knowledge in the human brain. Proc. Natl. Acad. Sci. USA.

[25] Lambon Ralph MA, Ehsan S, Baker GA, Rogers TT (2012). Semantic memory is impaired in patients with unilateral anterior temporal lobe resection for temporal lobe epilepsy. Brain.

[26] Jefferies E, Lambon Ralph MA (2006). Semantic impairment in stroke aphasia versus semantic dementia: a case-series comparison. Brain.

[27] Patterson K, Nestor PJ, Rogers TT (2007). Where do you know what you know? The representation of semantic knowledge in the human brain. Nat. Rev. Neurosci..

[28] Rogers TT, McClelland JL (2003). Semantic Cognition: A Parallel Distributed Processing Approach.

[29] Rabovsky M, McClelland JL (2020). Quasi-compositional mapping from form to meaning: a neural network-based approach to capturing neural responses during human language comprehension. Philos. Trans. R. Soc. Lond. Ser. B Biol. Sci..

[30] Price CJ (1994). Brain activity during reading.. The effects of exposure duration and task. Brain.

[31] Pugh KR (1996). Cerebral organization of component processes in reading. Brain.

[32] Lau EF, Phillips C, Poeppel D (2008). A cortical network for semantics: (De)constructing the N400. Nat. Rev. Neurosci..

[33] Cromer JA, Roy JE, Miller EK (2010). Representation of multiple, independent categories in the primate prefrontal cortex. Neuron.

[34] Roy JE, Riesenhuber M, Poggio T, Miller EK (2010). Prefrontal cortex activity during flexible categorization. J. Neurosci..

[35] Gardner HE (2012). The differential contributions of pFC and temporo-parietal cortex to multimodal semantic control: exploring refractory effects in semantic aphasia. J. Cogn. Neurosci..

[36] McKee JL, Riesenhuber M, Miller EK, Freedman DJ (2014). Task dependence of visual and category representations in prefrontal and inferior temporal cortices. J. Neurosci..

[37] Perret E (1974). The left frontal lobe of man and the suppression of habitual responses in verbal categorical behaviour. Neuropsychologia.

[38] Thompson-Schill SL (2003). Neuroimaging studies of semantic memory: inferring "how" from "where". Neuropsychologia.

[39] Jefferies E (2013). The neural basis of semantic cognition: converging evidence from neuropsychology, neuroimaging and TMS. Cortex.

[40] Buckner RL, Andrews-Hanna JR, Schacter DL (2008). The brain's default network: anatomy, function, and relevance to disease. Ann. N.Y. Acad. Sci..

[41] Corbetta M, Shulman GL (2002). Control of goal-directed and stimulus-driven attention in the brain. Nat. Rev. Neurosci..

[42] Amano S, Kondo T (1999). Nihongo-no Goitokusei: Lexical Properties of Japanese.

[43] Kreuz RJ (1987). The subjective familiarity of English homophones. Mem. Cogn..

[44] Amano S, Kondo T (2000). Nihongo-no Goitokusei: Lexical Properties of Japaneses.

[45] De Deyne S (2008). Exemplar by feature applicability matrices and other Dutch normative data for semantic concepts. Behav. Res. Methods.

[46] McRae K, Cree GS, Seidenberg MS, McNorgan C (2005). Semantic feature production norms for a large set of living and nonliving things. Behav. Res. Methods.

[47] Caramazza A, Hersh H, Torgerson WS (1976). Subjective structures and operations in semantic memory. J. Verbal Learn. Verbal Behav..

[48] Soshi T, Fujimaki N, Matsumoto A, Ihara SA (2017). Memory-based specification of verbal features for classifying animals into super-ordinate and sub-ordinate categories. Front. Commun..

[49] Kriegeskorte N, Simmons WK, Bellgowan PS, Baker CI (2009). Circular analysis in systems neuroscience: The dangers of double dipping. Nat. Neurosci..

[50] Iidaka T, Matsumoto A, Nogawa J, Yamamoto Y, Sadato N (2006). Frontoparietal network involved in successful retrieval from episodic memory. Spatial and temporal analyses using fMRI and ERP. Cereb. Cortex.

